# Challenges Facing Contemporary Interventional Cardiology

**DOI:** 10.31083/RCM50691

**Published:** 2026-07-16

**Authors:** Lloyd W Klein

**Affiliations:** ^1^Cardiology Division, Department of Medicine, University of California, San Francisco, CA 94117, USA

## 1. Introduction

Interventional cardiology (IC) has been among the most innovative medical specialties of the modern era. Few fields have had such an impact on patients’ lives, who benefit from improved survival and quality of life. Advances in vascular treatment devices and imaging modalities have transformed coronary artery disease into a condition that is often treated more rapidly and effectively.

Despite these contributions, IC faces a workforce crisis. Many IC fellowship positions went unfilled over the past two fellowship cycles in the United States. This signals that the profession is perceived to be less attractive to future cardiologists. Analyses from the Society for Cardiovascular Angiography and Interventions (SCAI) detail this trend [1,2].

In essence, the problem is that IC has been structured with an emphasis on hospital economics and an overproduction of workforce, which detracts from sustainability and morale [3,4,5]. The decreasing rates of professional reimbursement have further intensified the pressure to perform an increased volume of cases.

## 2. How Hospital Economics Restructured the Field

Hospitals have recognized that providing 24/7 percutaneous coronary intervention (PCI) capabilities is an economic driver. An ST-elevation myocardial infarction (STEMI) patient brings a cascade of revenue-generating care: emergency services, intensive care, imaging, pharmacy, laboratory testing, telemetry, rehabilitation, and downstream cardiology follow-up. Cardiovascular care remains one of the most profitable service lines in American medicine.

Consequently, the majority of hospitals pursue designation as STEMI centers. Even in areas where only two or three high-volume PCI hospitals are necessary, there may be five or six institutions vying to provide comprehensive interventional services. This growth is motivated more by the desire for market share than by the actual needs of the community.

But 24/7 PCI coverage requires interventional cardiologists to staff nights, weekends, and holidays. Unlike trauma surgery or anesthesia, hospitals have largely avoided paying explicitly for this service. Instead, the cost has been absorbed into professional expectations: the per-procedure reimbursement does not separately compensate emergency coverage obligations. Prior work from the ACC Interventional Section shows how acute care responsibilities now dominate contemporary practice [6].

## 3. On-Call Burden and Professional Sustainability

Modern interventional practice is characterized by continuous on-call responsibility. STEMI coverage is expected 24 hours a day, 365 days a year in most hospitals. Interventionalists, often early in their careers, are routinely tasked with substantial coverage for emergent cases. This demand carries a physical and psychological toll. Emergent PCI requires rapid judgment and technical precision, often in the setting of sleep deprivation and cumulative stress. While many specialties have moved toward more predictable workflows, IC remains anchored to a round-the-clock model that many young physicians find deterrent. The effects of acute care obligations on professional sustainability have been documented [6].

At the same time, procedural volume is being diluted across an expanding workforce. To staff multiple hospitals, systems have hired more interventional cardiologists, but acute case volume has not increased proportionally. Elective procedures are now spread across too many operators. Procedural mastery depends on repetition. When volume per operator falls, confidence and proficiency suffer.

## 4. A Market Signal the Profession Can No Longer Ignore

When IC entered the National Residency Matching Program (NRMP) Specialties Matching Service in 2024, the results were sobering. For the 2025 appointment year, 326 fellowship positions were offered, and roughly 54 went unfilled. In the following cycle, 307 positions were offered, but only 244 applicants entered the Match, leaving 63 positions vacant even though nearly all applicants matched. These findings were summarized in SCAI’s official response and town hall analysis [1,2].

That pattern is unusual for a historically competitive procedural subspecialty. It reflects a collective judgment by trainees that the professional price of entering IC may now exceed the potential rewards.

## 5. The Fellowship System as Labor Infrastructure

The transfer of costs to the physician is reinforced by the fellowship system itself. IC fellows are trainees, but they function as essential workers. They conduct evaluations, secure consent, oversee peri-procedural care, write notes and reports, address overnight concerns, and staff catheterization labs. They take on a significant portion of the operational responsibilities of interventional services at salaries comparable to residents. Hospitals incorporate fellows into their operations as a cost-efficient strategy that enables the availability of continuous service. Employing equivalent technical and attending-level personnel would necessitate substantial salaries, in the high-five- to six-figure range, along with benefits, which many programs find financially unfeasible. They facilitate lab turnover, allowing more cases to be done. Importantly, they protect attendings from the full intensity of the workload.

## 6. Declining Reimbursement

This crisis is unfolding simultaneously with a decrease in reimbursement for procedural performance. Medicare and commercial payers determine payment through relative value units (RVUs) and a conversion factor. Both are under pressure. Centers for Medicare & Medicaid Services (CMS) has reduced RVU valuations and cut practice-expense allocations for hospital-based services. Budget-neutrality rules have repeatedly lowered the conversion factor, so that even stable RVU production yields less real income [3,4,5].

The result is that interventional cardiologists receive lower compensation per case and reduced payment per unit of work (wRVU), despite the increasing complexity of cases and growing administrative demands. Hospitals react predictably: they increase their workforce to provide greater coverage, anticipating that this will lead to an increase in volume and, consequently, revenue. Meanwhile, the financial burden is borne by the workforce.

## 7. Consequences for Practice and Training

For practicing interventionalists, the system produces a paradox: relentless call obligations combined with shrinking elective volume per operator. Too many physicians are chasing too few cases, eroding technical mastery while creating exhaustion.

The U.S. currently has roughly 6700 certified interventional cardiologists, or 20.2 per million population [7]. The median number of PCI procedures per operator is 59 per year, reflecting the fact that 44% of U.S. operators perform fewer than 50 PCIs per year, which is the arbitrary threshold set by accrediting organizations [8]. Europe has a lower median density, with 13.5 ICs per million population across member countries [9]. In Japan, there are roughly 64 per million, with a skew toward urban centers; the median operator volume in Japan is 28 PCIs per year [10].

Performance continues to be judged by procedural volume rather than adherence to appropriate-use criteria and clinical judgment.

In a volume-driven system, patient selection can drift toward treating borderline indicated or very low risk cases to sustain procedural numbers, which changes the emphasis from benefit to volume. Operator selection may skew outcomes when lower-risk cases are concentrated among high-volume physicians, while higher-risk cases are avoided to protect their outcome metrics. Unless the use of these strategies is explicitly sought, performance data may reflect case mix and incentive structure more than true technical superiority or clinical judgment [11].

For those in training, the message is unmistakable. IC entails significant responsibility, considerable stress, diminishing financial compensation, and limited control over personal time. Although the field continues to require exceptional dedication and offers a highly fulfilling career, it no longer consistently provides the autonomy or stability that previously justified personal sacrifices. The unfilled fellowship slots may reflect this perception [1,2].

## 8. What Can Be Done

*Reduce Overproduction*. IC fellowship positions should be tied to national procedural volume. Training more interventionalists rather than meaningful coronary work can support increased call burden and incentives for marginal use. Accreditation standards must require and enforce minimum per-fellow and per-faculty case volumes [12,13]. Current fellowship standards require ≥250 cases (≥200 coronary interventions and ≥50 additional procedures), reflecting expert consensus rather than evidence directly linking experience with outcomes. Many programs do not consistently achieve these thresholds, which may impact readiness for independent practice [13,14].

*Regionalize STEMI Coverage*. Instead of every hospital staffing 24/7 PCI coverage, systems should adopt regional STEMI hubs, as in trauma and stroke care. This would concentrate cases and increase individual operator volume [6].

*Differentiate Emergency and Elective Tracks*. IC should formally recognize two paths: acute coronary interventionists and elective coronary specialists. These roles involve different skills and career paths and should be compensated and supported accordingly [7].

*Pay Explicitly for On-Call Coverage*. Hospitals currently shift the cost of STEMI coverage onto physicians. Call should be treated as a service line, with explicit payment or reduced RVU expectations. If hospitals bore the true cost of 24/7 PCI, coverage would consolidate, and physician time would be properly valued [3,4,5].

*Right-Size IC Manpower*. Each institution staffs ICs for procedures, ICU/CCU coverage, consults, clinics, and call, resulting in more operators than case volume supports. Medicare data have shown this mismatch between operator numbers and meaningful case volume [10]. The board certification minimum of 50 cases/year is consensus-based and not clearly tied to outcomes data [8].

## 9. Why Reform Is Difficult

The tragedy is that solutions do exist, but the system resists them. Reducing fellowship slots raises labor costs. Regional STEMI care reduces market share. Paying for a call exposes the true cost of coverage. Each reform shifts value away from institutions and back toward physicians.

No individual program can afford to act alone. Cutting slots means losing labor, increasing attending call, and weakening recruitment. The existence of several hundred IC training programs reflects institutional incentives that conflict with workforce demand [1,2].

## 10. Conclusion

IC is now confronted with a workforce paradox: a legacy of clinical achievements paired with diminishing trainee enthusiasm. The empty fellowship positions are not unexpected. They are the likely consequence of a system where workforce expectations and institutional incentives are not coordinated. Unless reforms are instituted, the field risks evolving into a low-volume, overstaffed procedural specialty. Training in coronary procedures will increasingly serve as a gateway to structural subspecialties until those, too, become oversaturated. When the workforce is utilized to optimize institutional profits, we put our clinical effectiveness and professional identity at risk.
